# Extraction of a Penetrating Nasocranial Foreign Body via Transnasal Endoscopic Approach

**DOI:** 10.1002/ccr3.71508

**Published:** 2025-11-20

**Authors:** Kinga Yo, Yasuhiro Takahashi, Ryoga Yo, Mariko Arimoto, Tessei Kuruma, Yasue Uchida, Yasushi Fujimoto

**Affiliations:** ^1^ Department of Otorhinolaryngology Aichi Medical University School of Medicine Aichi Japan; ^2^ Department of Oculoplastic, Orbital and Lacrimal Surgery Aichi Medical University Hospital Aichi Japan

**Keywords:** cerebral angiography, nasocranial foreign body, radiographical evaluation, skull base injury, transnasal endoscopic approach

## Abstract

A 42‐year‐old male presented to our hospital with right ocular pain, diplopia, and difficulty opening his right eye. Three days earlier, while pulling up a fishing rod, a sinker struck his face. Since then, he developed decreased vision in his left eye and visited a local ophthalmologist the following day, where a retinal hemorrhage in the left eye was diagnosed. Two days later, he developed dull pain in his right eye and revisited the ophthalmologist on the third day after the injury. A computed tomography (CT) scan revealed a foreign body in the paranasal sinus, and he was subsequently referred to our hospital. Upon initial examination at our hospital, complete ophthalmoplegia and right‐sided ptosis were observed. CT scan revealed a metallic foreign body in the paranasal sinus, penetrating the orbit and middle cranial fossa. Considering that the foreign body was a metallic sinker, magnetic resonance imaging (MRI) was not performed. Cerebral angiography revealed no direct injury to the internal carotid artery. The foreign body was extracted using a transnasal endoscopic approach and was confirmed to be a fishing sinker. Postoperatively, intravenous steroids and antibiotic treatment were administered. Ophthalmoplegia and ptosis completely resolved.

## Key Clinical Message

In nasocranial foreign body cases, careful preoperative radiographic assessment is essential to evaluate intracranial location and cerebral injury and to guide procedural safety. Delayed neurological deficits may be treatable with solely transnasal endoscopy if they are not caused by direct brain injury.

## Graphical abstract

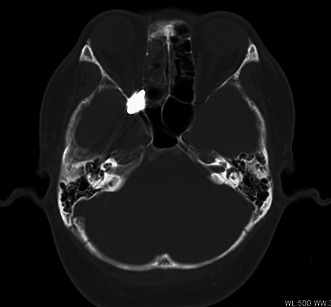

## 1 Introduction

Nasal foreign bodies are a condition that otolaryngologists often encounter in regular outpatient practice; however, intracranial penetration is rare, occurring in only 0.4% of head injuries [1, 2]. In such cases, craniotomy is typically required to remove the foreign body [3]. However, this invasive approach can cause neurological complications related to intracranial manipulation [4]. Recently, a transnasal approach has been reported as a less invasive method to remove nasocranial foreign bodies [5]. In cases presenting with delayed‐onset neurological deficits, careful preoperative imaging evaluation enables effective treatment planning. Herein, we present a case of a nasal foreign body penetrating the cranial cavity, highlighting the feasibility and effectiveness of a transnasal endoscopic approach as a therapeutic option. This case report also emphasizes the importance of a preoperative radiographic evaluation of the patient's condition.

## 2 Case Report

### 2.1 Presenting Complaints and Disease History

A fishing sinker struck the right eye of a 42‐year‐old man and was lost when the fishing rod was pulled up. The chief complaint was trauma to the right eye; however, on the day of the ocular trauma, an ophthalmologist diagnosed the patient with a left retinal hemorrhage. However, 3 days later, the patient developed diplopia and right‐sided ptosis.

### 2.2 Differential Diagnosis, Investigations and Treatment

**FIGURE 1:**
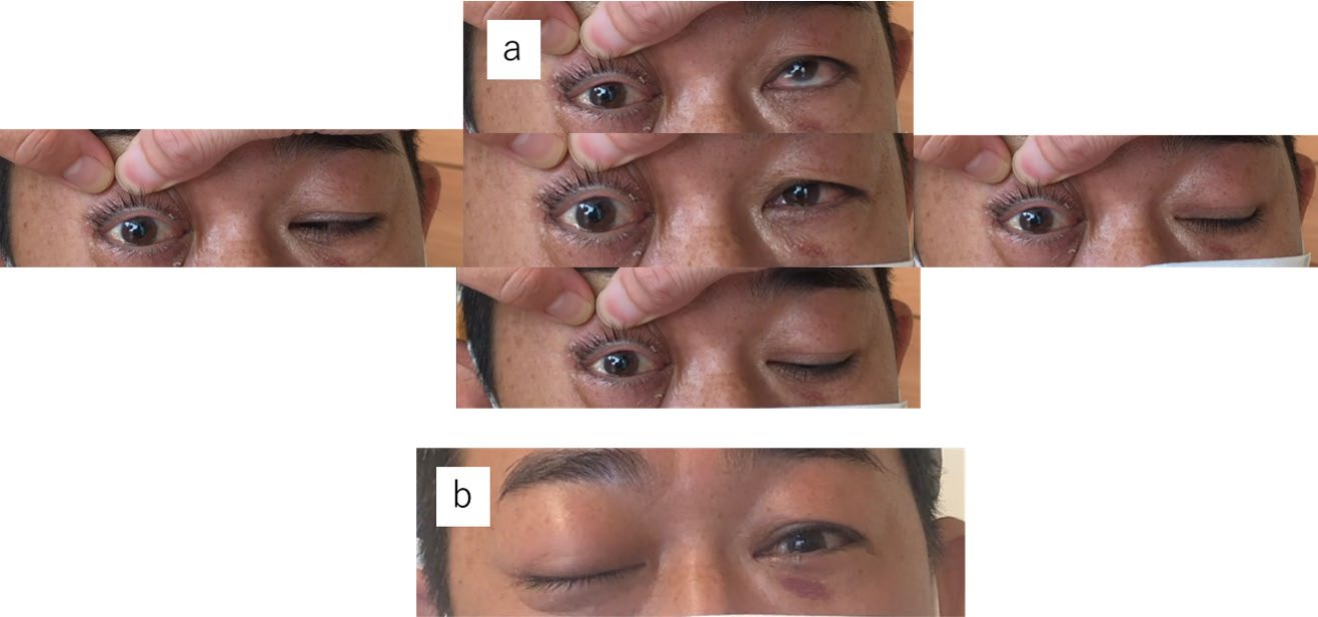
Facial photographs taken at the first examination. The right eye is fixed in the primary position with no movement medially, laterally, upward, or downward (a). Ptosis is also observed (b).

**FIGURE 2:**
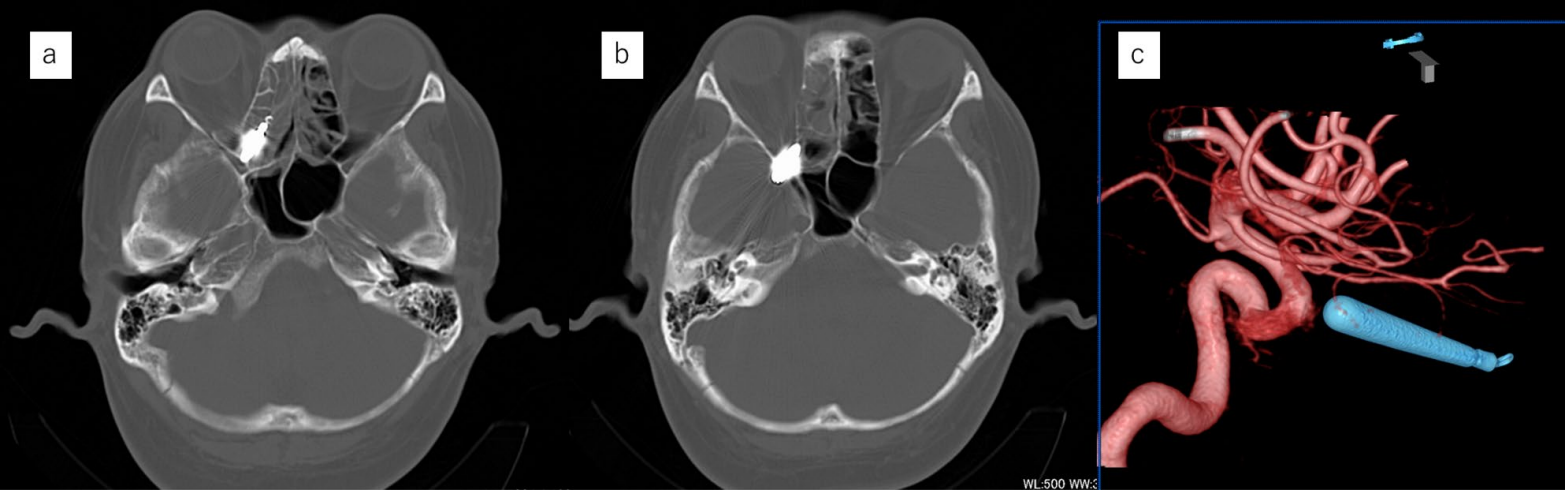
Computed tomography (CT) images. (a and b) Axial CT images showing a foreign body located in the paranasal sinus and penetrating the orbit and middle cranial fossa. (c) Cerebral angiography showing the foreign body located away from the internal carotid artery.

**FIGURE 3:**
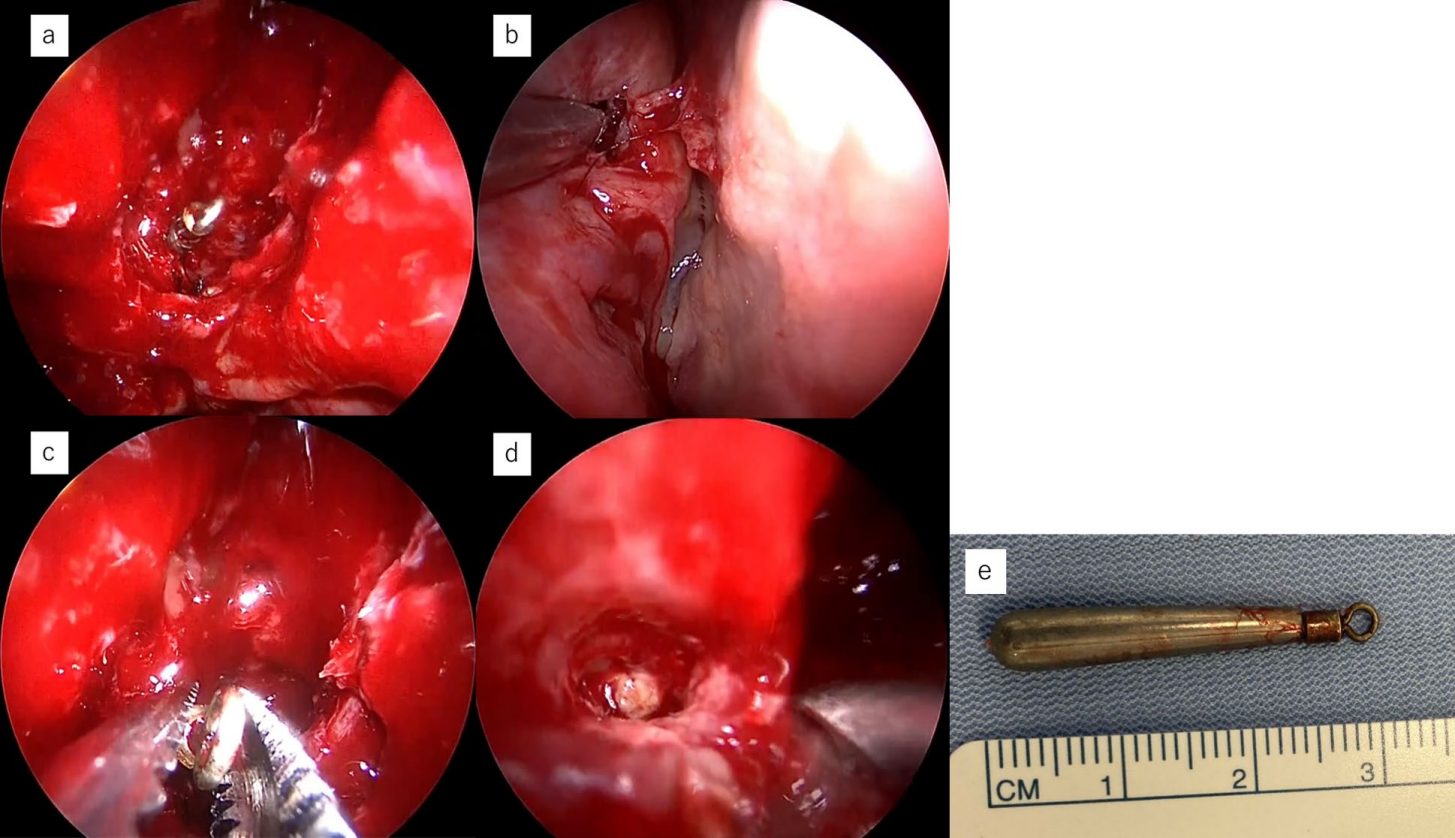
Intraoperative nasal endoscopic images. (a) Following full exposure of the right paranasal sinus, the posterior end of the foreign body was visualized. (b) Perforation of the cephalic side of the superior meatus indicated the foreign body's trajectory. (c) The foreign body was grasped and extracted. (d) No bleeding or cerebrospinal fluid leakage was observed at the surgical site. (e) The extracted foreign body was confirmed to be a tungsten fishing sinker approximately 3 cm in length.

On initial examination in the emergency department, 4 days after the trauma, complete ophthalmoplegia and right‐sided ptosis were observed (Figure 1a,b). Computed tomography (CT) revealed a metallic foreign body in the right posterior ethmoid sinus, penetrating the middle cranial fossa through the inferior and superior orbital fissures. The brain did not appear to be directly injured (Figure 2a,b), and there was no brain hemorrhage. The nasal septum was deviated in an inverted S‐shape in the anteroposterior direction. No nasal bone fracture was observed, and the nasal septal deviation was determined not to be caused by the current trauma. Cerebral angiography revealed a foreign body located away from the internal carotid artery (Figure 2c). The patient was admitted to the hospital the same day and was administered intravenous antibiotics. Meropenem at 1 g/day was administered for 1 week to prevent meningitis. Two days after admission, the foreign body was extracted via a transnasal endoscopic approach under general anesthesia, with neurosurgeons on standby. Upon opening the right posterior ethmoid sinus, the foreign body was observed entering the inferomedial orbit (Figure 3a). The cephalic side of the superior meatus was perforated, implying the path of the foreign body (Figure 3b). The ethmoid sinus, maxillary sinus, frontal sinus, and sphenoid sinus were opened, and, after complete exposure of the intranasal portion of the foreign body, the sinuses were carefully cleared (Figure 3c,d). To prevent intracranial injury and cerebrospinal fluid fistula, care was taken to avoid manipulating the dura mater until the final stage of excision of the sinker, and the paranasal sinuses were widely opened to ensure adequate space for manipulation in the event of complications. There was no evidence of intraoperative bleeding or cerebrospinal fluid fistula. The extracted foreign body was found to be a tungsten fishing sinker approximately 3 cm in length (Figure 3e).

### 2.3 Outcome and Follow‐Up

**FIGURE 4:**
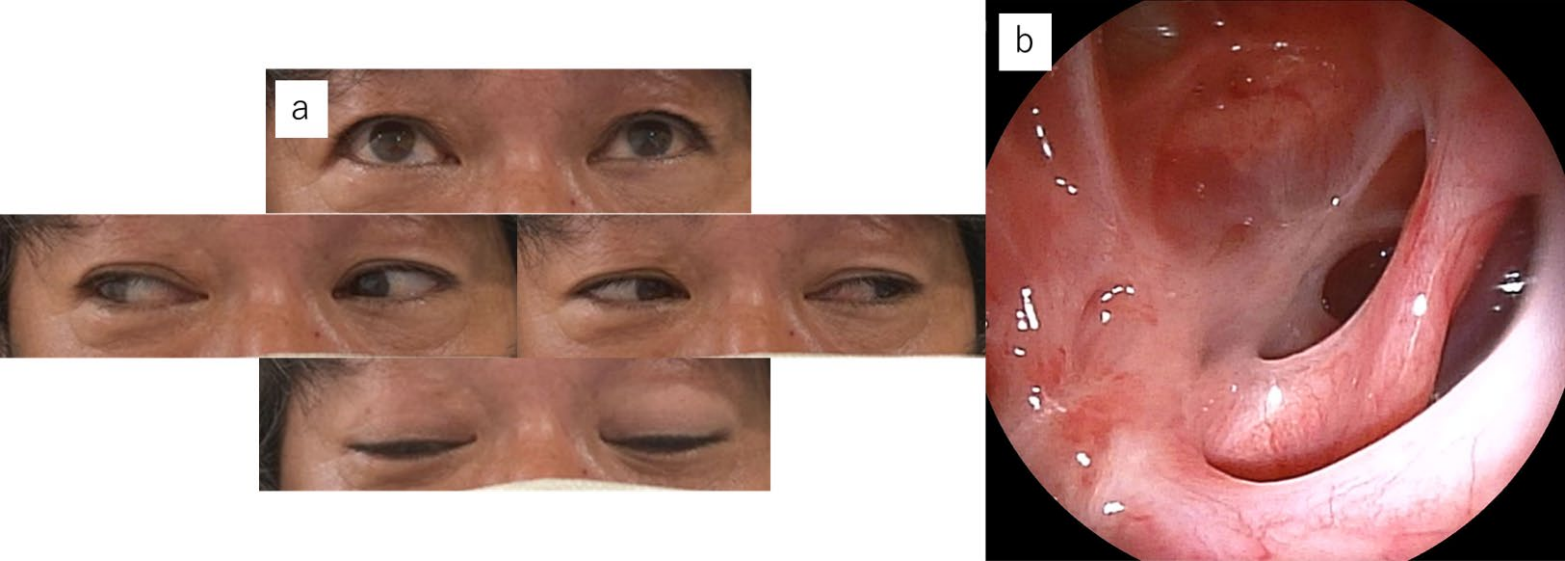
Images obtained 5 weeks after surgery. (a) Resolution of ophthalmoplegia. (b) Clean surgical site in the paranasal sinus.

After surgery, the patient received 3 cycles of 500 mg/day of intravenous hydrocortisone for 3 days/week for neuroprotection. Five weeks postoperatively, ophthalmoplegia and ptosis had completely resolved (Figure 4a,b).

## 3 Discussion

In the present case, a nasal foreign body penetrating the orbit and middle cranial fossa was successfully removed using a transnasal endoscopic approach. Postoperatively, the diplopia and ptosis resolved. Although the transnasal endoscopic approach is less invasive than a craniotomy for the removal of intracranial foreign bodies, it should be indicated only in selected cases [6, 7]. Suggested indications for the transnasal endoscopic approach are as follows: absence of brain hemorrhage, no parenchymal or vascular damage, no neurological deficits, the surgeon's ability to repair cerebrospinal fluid leaks via the same approach, and preparation of a neurosurgical team for critical complications [8]. In this case, the preoperative CT scan revealed no brain hemorrhage or parenchymal damage. No direct injury to the internal carotid artery was confirmed by cerebral angiography. Although this patient exhibited diplopia and ptosis, he noticed these symptoms 3 days after the injury. This delayed onset suggested an inflammatory response around the foreign body rather than direct damage to the oculomotor nerve [9, 10]. Rapid improvement in ocular symptoms after steroid treatment is consistent with this inflammatory mechanism. These findings confirm the feasibility of using the transnasal endoscopic approach alone to remove foreign bodies. No serious intracranial complications were encountered during surgery.

The absence of direct injuries to the cerebral parenchyma and internal carotid artery in this case may be related to the nasal septal deviation. The anterior portion of the nasal septum deviated toward the left nasal cavity, whereas the posterior portion curved superiorly. The foreign body may have rebounded from the nasal septum and been redirected toward the orbital side. At that time, its kinetic energy might have been dissipated by the ethmoid air cells and attenuated after colliding with the orbital wall, preventing penetration of the cerebral parenchyma and the internal carotid artery. The blunt tip of the foreign body may also have prevented deep intracranial penetration.

## 4 Conclusion

We present a case of a nasocranial foreign body that was successfully extracted using a transnasal endoscopic approach. Preoperative radiographic evaluation of the intracranial location of the foreign body and the presence or absence of cerebral damage provide critical information on the feasibility of the endonasal–transnasal approach and risk of serious cerebral complications. In cases of delayed onset neurological deficits, if the deficits are not caused by direct injury, endoscopic treatment may be possible, and neurological symptoms can also be treated.

## Author Contributions

**Kinga Yo:** conceptualization, data curation, methodology, project administration, resources, supervision, visualization, writing – original draft. **Yasuhiro Takahashi:** data curation, writing – review and editing. **Ryoga Yo:** data curation, investigation. **Mariko Arimoto:** writing – review and editing. **Tessei Kuruma:** writing – review and editing. **Yasue Uchida:** writing – review and editing. **Yasushi Fujimoto:** writing – review and editing.

## Ethics Statement

As a single‐case report with the patient's signed consent, no other ethical review was required.

## Consent

Written informed consent was obtained from the patient for the publication of this case report.

## Conflicts of Interest

Yasuhiro Takahashi received speaker and consultant honoraria from Amgen Inc., Chugai Pharmaceutical Co. Ltd., Santen Pharmaceutical Co. Ltd., and Ono Pharmaceutical Co. Ltd. The other authors have no financial or conflicts of interest to disclose.

## Data Availability

The data that support the findings of this study are available on request from the corresponding author. The data are not publicly available due to privacy or ethical restrictions.
